# Filovirus vaccines as a response paradigm for emerging infectious diseases

**DOI:** 10.1038/s41541-024-00985-y

**Published:** 2024-10-11

**Authors:** Andrea Marzi, Heinz Feldmann

**Affiliations:** Laboratory of Virology, Division of Intramural Research, National Institute of Allergy and Infectious Diseases, National Institutes of Health, Rocky Mountain Laboratories, Hamilton, MT USA

**Keywords:** Microbiology, Ebola virus

## Abstract

Nowadays, filovirus vaccine development may be seen as a paradigm for our response capabilities to emerging and re-emerging infectious diseases. Specifically, the West African Ebola virus disease (EVD) epidemic accelerated countermeasure licensure for several vaccine and therapeutic products. Those products have been successfully used to control EVD outbreaks in Central Africa over the past years. This positive development, however, has not yet reached beyond EVD. Therefore, it is pertinent to increase our efforts in the development of countermeasures for other human pathogenic members of the family *Filoviridae* as they continue to threaten public health in Sub-Saharan Africa. This review article summarizes the current filovirus vaccines in preclinical macaque studies and human clinical trials and discusses the most promising recent advancements.

## Introduction

Filoviruses are a family of enveloped, non-segmented, negative-strand RNA viruses and are mainly endemic to Africa. The family is divided into several virus genera, but outbreaks of human disease have only been caused by ebolaviruses and marburgviruses^1^. Infection with the most prominent members—Ebola virus (EBOV) or Marburg virus (MARV)—causes hemorrhagic disease with case fatality rates of up to 90%^1^. Outbreaks of human disease are believed to originate from zoonotic spillover events from the reservoir, likely bats, or other end host species such as nonhuman primates (NHPs), both sources of nutrition in the endemic areas. Bats are the reservoir of MARV^2^ and have long been speculated to be the reservoir for ebolaviruses^1^. EBOV and MARV recently caused disease outbreaks in areas outside of their known endemic zones which may be attributed to an expanding interface between reservoir species and the human host driven by changes in the environment, economy, public health and human behavior. Human-to-human transmission through persistently infected survivors has been suspected for a long time and recently been confirmed; however, its contribution to causing future outbreaks remains unclear but a worrying probability^3,4^.

The 2013–2016 West African EBOV disease (EVD) epidemic accelerated countermeasure development and licensure for several products specific to EBOV in the diagnostic, vaccine, and treatment sector. As a result, two EBOV vaccines—Ervebo (based on vesicular stomatitis virus (VSV); also known as VSV-EBOV or rVSV-ZEBOV)^5^ and the prime-boost combination of Zabdeno (based on adenovirus (Ad); also known as Ad26-EBOV) and Mvabea (based on modified vaccinia Ankara (MVA); also known as MVA-BN-filo)^6^—have been approved for human use by US, European and African authorities (Table 1). In recent years, these vaccines along with the monoclonal antibody-based therapeutics Ebanga (also known as ansuvimab; single monoclonal antibody)^7^ and Inmazeb (also known as REGN-EB3; cocktail of 3 monoclonal antibodies)^8^ have been successfully used to combat EVD outbreaks in the Democratic Republic of the Congo (DRC)^9^. However, these approved vaccines and treatments are specific to EBOV with limited to no use against other ebolaviruses and MARV^10^. It is therefore pertinent to continue the development of countermeasures for other ebolaviruses, like Sudan virus (SUDV) and Bundibugyo virus (BDBV), and MARV as they continue to cause human disease outbreaks albeit with lower case fatality rates (SUDV ~50%, BDBV ~33%)^11^ and pose a threat to public health in Africa^12^.

**Table 1:** Summary of species-specific filovirus vaccines with preclinical efficacy in NHP models

Vaccine	Target Species	Antigen used	Approach	Status	Product	Comments
VSV-EBOV & Ad5-EBOV	EBOV	EBOV-Makona GP	Prime-boost	Licensed in Russia; 2015	GamEvac-Combi	Largely limited to use by Russia
Ad5-EBOV	EBOV	EBOV-Makona GP		Licensed in China; 2017		Largely limited to use by China
VSV-EBOV	EBOV	EBOV-Kikwit GP	Prime	Licensed in USA, Europe, Africa; 2019	Ervebo	In use and recommended by WHO
Ad26-EBOV & MVA-BN-filo	EBOV	Ad26: EBOV-Mayinga GP MVA: EBOV-Mayinga GP SUDV-Gulu GP MARV-Musoke GP TAFV NP	Prime-boost	Licensed in Europe, Africa; 2020	Zabdeno & Mvabea	In use and recommended by WHO
cAd3-SUDV	SUDV	not disclosed		Clinical trials, phase I		Clinical development supported by BARDA
VSV-SUDV	SUDV	SUDV-Boneface GP	Prime	Preclinical		International AIDS Vaccine Initiative
ChAdOx-biEBOV	EBOV & SUDV	EBOV-Makona GP & SUDV-Boneface GP		Clinical trials, phase I		Development ceased
VSV-BDBV	BDBV	BDBV GP	Prime	Preclinical		Currently not a priority
VSV-TAFV	TAFV	TAFV GP	Prime	Preclinical		Not a priority – only a single non-lethal human case known
VSV-MARV	MARV	MARV-Angola GP	Prime	Clinical trials, phase I		Public Health Vaccines
VSV-MARV	MARV	MARV-Musoke GP	Prime	Preclinical		International AIDS Vaccine Initiative
VSV-N4CT1-MARV	MARV	MARV-Angola GP	Prime	Preclinical		Auro Vaccines
cAd3-MARV	MARV	MARV-Angola GP	Prime & prime-boost	Clinical trials, phase I		Sabin Vaccine Institute

Several vaccine platforms have been explored harnessing their advantages for rapid onset of protection and/or durability of the protective immune response. VSV-based vaccines are live-attenuated viral vectors administered as single-dose vaccines known to elicit protective immunity within 7–10 days of vaccination^13–15^. This vaccine platform has also demonstrated limited post-exposure efficacy in NHPs for filoviruses^16,17^. The cAd3 vaccines have also demonstrated fast-acting potential in NHPs, however, no confirmative human data are yet available^18^. Other platforms used to develop multi-dose approaches include protein-based subunit vaccines^19^ and non-replicating viral vectors including heterologous prime/boost candidates^20^ known to elicit durable immunity.

Preclinical efficacy studies in a variety of animal models are critical for the clinical development and licensure of filovirus vaccines. While rodent models (mice, hamster and Guinea pigs) are excellent screening models for filoviruses, ultimately NHP studies are needed to establish the protective efficacy of a filovirus vaccine^21^. This fact was recently highlighted when a bivalent EBOV and SUDV vaccine achieved great immunogenicity in NHPs and humans yet failed to provide any protection against lethal SUDV challenge in the macaque disease model^22^.

Increased surveillance and advanced sequence technologies have resulted in the discovery of novel filoviruses allowing for virus isolation (Lloviu virus, LLOV)^23^ or generation of recombinant viruses using reverse genetics systems (Bombali virus, BOMV)^24,25^. However, little is known about the pathogenic potential of these bat-derived viruses in other species including humans complicating countermeasure development and highlighting a knowledge gap that needs to be addressed.

With the approval of EBOV vaccines and therapeutics, the focus in the filovirus countermeasure field has shifted to SUDV and MARV^26^ as well as pan-filovirus approaches to enable a quick public health response to control filovirus disease outbreaks. This review summarizes the current state of filovirus vaccine development with a focus on preclinical macaque and human clinical trial data for vaccine candidates with trajectory towards approval.

### EBOV vaccines

Globally, there are currently 4 EBOV vaccines which were approved by regulatory authorities in the wake of the 2013–2016 West African EVD epidemic. Russia was the first country to approve a heterologous prime-boost vaccine consisting of VSV-EBOV (based on the VSV glycoprotein-deleted platform, VSVΔG) and Ad5-EBOV (GamEvac-Combi) in 2015 for emergency use. Then, in 2017, China approved its Ad5-based EBOV vaccine^27^. Finally, in 2019, the European and US authorities approved two vaccines, VSV-EBOV and the prime-boost combination Ad26-EBOV/MVA-BN-filo^28^. Approvals for both vaccines have recently been updated to include children 12 months and older in addition to adults^28,29^. Numerous countries in Africa and the WHO have approved the use of the latter two vaccines for broader population-based vaccination with the VSV-EBOV vaccine also authorized for use as an emergency vaccine during outbreaks^30,31^. This vaccine is fast-acting in NHPs^13^ and humans^14^ and has recently been shown to indeed reduce mortality by 50% in vaccinated people during outbreaks^32^. EBOV vaccine development did not cease with the approvals of the above products as it remains the main cause of filovirus disease outbreaks and is included in all multivalent or pan-filovirus vaccine efforts discussed below.

### SUDV vaccines

The 2022 SUDV outbreak in Uganda^33^ brought this virus into the spotlight of filovirus research and particularly countermeasure development. A WHO panel ranked the available vaccines in the following order of priority: the VSV-SUDV (based on the VSVΔG platform), the chimpanzee Ad (cAd or ChAd)-based SUDV-specific vaccine cAd3-SUDV, and the ChAd-based EBOV/SUDV bivalent vaccine, ChAdOx-biEBOV^12^. Clinical-grade vaccine doses of ChAdOx-biEBOV, cAd3-SUDV, and VSV-SUDV were shipped to Uganda for emergency use under a clinical trial protocol^34^ and arrived ~80 days after the outbreak was declared^12^. Despite all these efforts, the international response was too slow, and the outbreak ended before vaccinations could be implemented. Critique with the response to this outbreak has been voiced^35^ including the need for faster vaccine deployment in preparation for future outbreaks. Of the three vaccines shipped to Uganda in 2022, there are two vaccine platforms ahead in development: cAd3-SUDV and VSV-SUDV. The ChAdOx-biEBOV vaccine did not demonstrate a protective benefit in a recent preclinical NHP study and development has ceased^36^. However, a phase 1 clinical trial was conducted (NCT05079750) with data not yet being released. The cAd3-SUDV vaccine has been shown to protect macaques from lethal SUDV challenge^37^ and is immunogenic with an acceptable safety profile at doses of 1 × 10^10^ and 1 × 10^11^ particle units (PU) in a recently published phase 1 clinical trial (NCT04041570)^38^. The single dose vaccination elicited antigen-specific antibodies in 78% of clinical trial participants within 2 weeks of vaccination and were durable up to 48 weeks. Neutralizing titers and T cell responses were also detected during the trial in most participants^38^. The VSV-SUDV vaccine protects NHPs within 4 weeks when administered by itself or in combination with other VSV-based vaccines^39,40^. Currently, this vaccine is in clinical development. Finally, there is also an adjuvanted SUDV GP-based subunit vaccine in development which is protective as a fresh and thermostable formulation after 3 doses in macaques^19,41^. This vaccine is moving towards clinical development.

### BDBV vaccines

BDBV is the latest of the human-pathogenic ebolaviruses. It was identified in the Bundibugyo district in Western Uganda following an outbreak of hemorrhagic disease in 2007 in humans with a case fatality of ~40%^42,43^. In 2012, BDBV re-emerged in the DRC causing an outbreak with ~54% case fatality rate^44^. Early after the initial outbreak, a macaque study showed some yet limited cross-protection against BDBV with VSV-EBOV or VSV-Taï forest virus (TAFV)^45^. This study was challenging to interpret, as BDBV infection is not uniformly lethal in macaques—a contrast to other filoviruses. In 2013, a VSV-BDBV vaccine expressing the BDBV GP was developed on the VSVΔG platform and tested in macaques. A single dose of this vaccine was shown to uniformly protect macaques from BDBV infection and lethal disease^46^. Even though this virus has not re-emerged, vaccine development should continue as BDBV is known to be pathogenic for humans.

### TAFV vaccines

Associated with only a single, non-fatal human case of disease contracted in Côte d’Ivoire, efforts in studying TAFV have been limited since its discovery in 1994^47^. However, with several filovirus outbreaks occurring in West Africa in the recent decade^33,48^, TAFV is of differential diagnostic importance and remains a pathogenic filovirus in humans. VSV-TAFV (based on the VSVΔG platform) is the only vaccine studied for protective efficacy in macaques against TAFV challenge so far^49^. A single dose was shown to be uniformly protective against lethal TAFV challenge with the vaccinated macaques not developing any signs of disease or viremia^50^. As stated above, this vaccine had been shown to provide limited cross-protection against BDBV infection^45^. The VSVΔG platform vaccines had previously demonstrated cross-protection against TAFV infection when a 1:1:1 blend of 3 VSV vaccines (VSV-EBOV, VSV-SUDV and VSV-MARV) was administered 4 weeks before lethal challenge. Uniform protection was achieved in the VSV blend group compared to 40% in the control group^40^. TAFV GP antibodies have also been demonstrated with Ad26 and Ad35 vaccination blending vectors expressing multiple filovirus GPs, however, no TAFV challenge was performed^51^. Lastly, MVA-BN-filo vector expresses the TAFV NP in combination with the SUDV GP, EBOV GP, and MARV GP but has never been evaluated against lethal TAFV challenge^52^. The cross-protective potential of this vaccine vector in combination with Ad26 vectors is discussed below.

### MARV vaccines

While MARV was the first filovirus to be discovered in 1967, it has not caused as many outbreaks of human disease compared to EBOV^53^. The two major outbreaks occurred in the Democratic Republic of Congo from 1997–2000 (154 cases, 128 fatalities) and Angola 2004–2005 (252 cases, 227 fatalities), yet MARV has not been as well-studied as EBOV^53^. This is also reflected in the progress made in countermeasure development. To date, there are 6 MARV-specific vaccines in preclinical and clinical development—one subunit vaccine, 3 vaccines based on the VSV platform, and 2 on the Ad platform. The subunit vaccine is composed of MARV GP and CoVaccine HT adjuvant and has demonstrated uniform protection in macaques after 3 doses^19^. This vaccine does not confer a rapid onset of protection, but the protective responses may be more durable. The VSV-N4CT1-MARV from Auro vaccines, a genetically altered VSV platform including the VSV glycoprotein, expresses the MARV-Angola GP as an additional open reading frame leading to uniform protection when a single high dose is administered prior to lethal MARV challenge. If this vaccine is administered closer to challenge (5 or 3 days prior), efficacy drops to 80% or 20% survival, respectively^54^. This is in contrast to VSV-MARV (expressing the MARV-Angola GP on the VSVΔG platform) for which vaccination with a single high dose even 3 days prior to infection leads to 75% survival in lethally MARV challenged macaques^15^. This vaccine is highly immunogenic and protects by eliciting a humoral as well as cellular responses^55^. Recently, this vaccine demonstrated uniform protection at a low dose within 7 days^56^, and is currently in clinical development by Public Health Vaccines with clinical trial data pending (NCT06265012)^26^. The last VSV-based MARV vaccine is the original version of VSV-MARV expressing the MARV-Musoke GP in the VSVΔG platform which demonstrated uniform protection in macaques within 28 days^10^ and durability of the uniformly protective response up to 407 days^57^. This vaccine is in clinical development by the International AIDS Vaccine Initiative^26^.

The 2 non-replicating viral vector vaccines are based on ChAd/cAd, namely ChAdOx-MARV (Oxford University) and cAd3-MARV (Sabin), expressing the MARV-Angola GP. There is no published data for the ChAdOx-MARV vaccine, but the cAd3-MARV has been shown to offer rapid (7 days before challenge) and durable (6 months before challenge) uniform protection in macaques with a single high dose^18^. After 12 months, the survival rate in single dose vaccinated macaques drops to 75%^18^. The cAd3-MARV is the only vaccine with available human clinical trial data. In a phase 1 trial in the USA the vaccine was shown to be immunogenic with an acceptable safety profile at doses of 1 × 10^10^ and 1 × 10^11^ PU (NCT03475056)^58^. Of the 40 participants 95% developed MARV GP-specific IgG 4 weeks after vaccination and the response remained measurable in 70% of the participants at 48 weeks^58^. A phase 2 clinical trial is currently ongoing in Uganda (NCT05817422).

### Multivalent filovirus vaccines

Since several human-pathogenic filoviruses overlap in their endemic areas of West, Central and East Africa, there is a push towards the development of pan-filovirus vaccines. Currently, there are several platforms in development for multi-valent approaches.

Bivalent subunit vaccines combining the insect cell-expressed GPs of EBOV and SUDV or EBOV and MARV with the adjuvant CoVaccine HT have been shown to uniformly protect macaques from lethal EBOV, SUDV, and MARV challenge after 3 doses^19^. In addition, the adjuvanted SUDV GP and MARV GP combination has uniformly protected SUDV- or MARV-challenged macaques after 3 vaccine doses^41^. Both formulations of these bivalent vaccines do not confer a rapid onset of protection, however, they are suitable for population-based vaccination particularly in the thermostable formulation. As adjuvanted subunit vaccines, adverse events are expected to be limited, however, the required 3 doses for protective efficacy presents a challenge for roll-out in rural filovirus endemic areas.

As stated above, while presumably immunogenic in a phase I clinical trial, a ChAdOx-biEBOV expressing both the EBOV GP and SUDV GP was not protective against lethal SUDV infection in macaques and development has ceased^36^. Based on the Ad26 platform, a trivalent filovirus vaccine consisting of Ad26-filo (a 1:1:1 blend of Ad26-EBOV, Ad26-SUDV and Ad26-MARV) and MVA-BN-filo has been shown to protect as a prime/boost strategy against lethal SUDV and MARV challenge in NHPs^52^. Notably, when MVA-BN-filo was administered first and Ad26-filo used as a boost, the protective efficacy decreased^52^. The safety and immunogenicity of doses as high as 9 × 10^10^ PU of Ad26-filo and 5 × 10^8^ infectious units of MVA-BN-filo administered 14 or 56 days apart was evaluated in a phase 1 clinical trial (NCT02860650). The study reports no serious adverse events after vaccination and 21 days after the boost vaccination all study participants had measurable binding antibody serum titers to the 3 filovirus GP antigens^20^. Neutralization activity was robust for EBOV, but suboptimal for SUDV and MARV^20^. This vaccine has not yet been moved further along in clinical development.

The VSV platform has also been explored for mediating pan-filovirus immunity. In 2009, single-dose vaccination with a blend of VSV-EBOV, VSV-SUDV and VSV-MARV provided uniform protection to macaques challenged with EBOV, SUDV, MARV or the heterologous TAFV^40^. However, no further studies have followed up on this concept using the VSVΔG vector. In contrast, Auro vaccines developed their VSV vector (VSV-N4CT1 including the VSV glycoprotein) into a trivalent formulation by blending vectors expressing EBOV GP, SUDV GP and MARV GP in a 1:1:1 ratio. A single dose protected all macaques from lethal challenge with EBOV, SUDV, or MARV^59^. Because filoviruses share their endemic areas with Lassa virus (LASV), the next study examining protection included a VSV vector expressing the LASV GP in the previously described trivalent blend. Administration of 2 doses (56 days apart) of this tetravalent blend (1:1:1:1 ratio) was uniformly protective against lethal challenge with EBOV, SUDV, MARV, and LASV in macaques^60^. Most recently, a study investigating the rapid onset of protection using a pan-filovirus vaccine (1:1:1:1 blend of VSV-N4CT1-EBOV, VSV-N4CT1-SUDV, VSV-N4CT1-MARV, and VSV-N4ΔG-BDBV) reported uniform protection within 7 days of vaccination for macaques challenged with SUDV, BDBV and MARV; however, EBOV challenge resulted in only 80% survival^61^.

### Future challenges

Filovirus vaccine development has generated licensed products for EVD but products for other human pathogenic filoviruses are not even close to the finish line. Efforts are being intensified to bring the most promising products, particularly for SUDV and MARV, from preclinical to clinical development and to licensure to cover potential reemergence in the African endemicity zones. These efforts include several new vaccine approaches based on updated platforms and new technologies which have yet to demonstrate preclinical efficacy in NHPs. In addition, mediation strategies need also be implemented for truly emerging, novel human-pathogenic filoviruses. As the development of these countermeasures is dependent on the continued infectious disease and public health interest and funding, efforts communicating these needs and bringing them to the stakeholders’ attention is critical.

Vaccine production for clinical use and vaccine storage for emergency release during outbreaks are challenging issues. The most effective way would likely be production in a developed country to use existing knowledge and facilities and storage in endemic regions for immediate and hopefully less complicated release in case of outbreaks. Currently largely unanswered questions relate to shelf-life time, storage temperature, and cold chain requirements of the different products. Continuation of these efforts requires further studies and appropriate funding.

Cross-protective vaccines would be helpful to cover the variation among human pathogenic filoviruses. However, single cross-protective vaccines would be preferred but are currently not available. Currently, cross-protective approaches include heterologous prime-boost and blending of vaccine candidates. Preclinical cross-protection studies may be difficult to interpret as protective efficacy is easier demonstrated in rodent than macaque models^62^. Taking this a step further, an extrapolation of cross-protective responses from macaques to humans is complicated as animal models use artificial exposure routes including intramuscular and intraperitoneal while natural filovirus infection is believed to occur mucosally^1^. Recent studies demonstrating disease in macaques after mucosal exposure are aiding in addressing this knowledge gap^63,64^.

Except for MVA-BN-filo, currently all filovirus vaccines, approved or in development, use the GP as the viral immunogen. Other viral proteins have been evaluated as immunogens especially for EBOV but with limited success^65^. Data from existing studies mapping immune responses to epitopes and other insightful details may inform the design of antigens especially those suitable to elicit cross-protective immune responses.

Finally, a rapid response strategy for vaccine development would be needed to address truly novel emerging filoviruses. Ideally, this would be based on existing successful vaccine platforms. To realize this effort, uncomplicated and immediate sharing of infectious material or virus isolates would be a prerequisite but is currently often not achievable.

## Conclusion

**Fig. 1:**
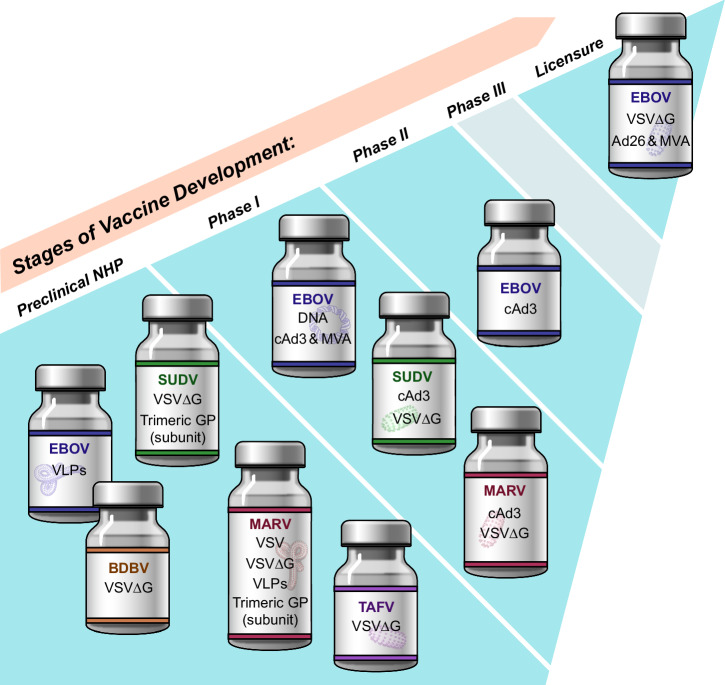
Development stages of filovirus vaccines in the USA. The cartoon indicates the development stages of different filovirus vaccines. Credit: NIAID.

Since the 2013–2016 West African EVD epidemic the filovirus field has come a long way with approved diagnostic tests, vaccines, and treatments against EBOV. Efforts are ongoing to reach a similar status with approved countermeasure for SUDV and MARV. There are several approaches in clinical development with promising initial results particularly on the vaccine front and the most recent outbreaks have revived interest and enabled funding^12^. These efforts should be expanded to include BDBV and TAFV as they remain the only known human-pathogenic filoviruses without a countermeasure landscape in considerable stages of development (Fig. 1). Along the same lines, studies should be intensified to define the pathogenic potential of novel filoviruses.

While the field is moving into the right direction, one of the biggest challenges remaining is the timeliness of the public health response after outbreaks are declared. Agreements are needed between countermeasure stakeholders, regulatory authorities, and governments to establish a framework particularly in the endemic areas in Africa to enable a faster public health response and deployment of vaccines and treatments to control disease outbreaks. A prerequisite for success is the involvement of African countries and their public health infrastructure.

Finally, lessons learned from filovirus vaccine development may guide responses to other infectious disease outbreaks, especially those caused by zoonotic pathogens. Response strategies, however, must be tailored to the individual pathogen/disease and there is no universal protocol that fits all. For instance, outbreaks caused by the likely bat-borne filoviruses are largely driven by human-to-human transmission, whereas outbreaks driven by the rodent-borne orthohantaviruses and mammarenaviruses are largely driven by rodent-to-human and only limited human-to-human transmission. Yet, implementations of vaccines can follow an analogous pathway for which EBOV can serve as a response paradigm. Therefore, we should ensure that preclinical development of vaccines for prioritized pathogens is accomplished including clinical grade material production, product storage, and arrangements for instantaneous, uncomplicated deployment. This will allow emergency clinical assessment during outbreaks similar to what was done in West African during the EVD epidemic.

## References

[1] Jacob, S. T. et al. Ebola virus disease. *Nat. Rev. Dis. Prim.* **6**, 13 (2020). 32080199 10.1038/s41572-020-0147-3 PMC7223853

[2] Towner, J. S. et al. Isolation of genetically diverse Marburg viruses from Egyptian fruit bats. *PLoS Pathog.* **5**, e1000536 (2009). 19649327 10.1371/journal.ppat.1000536 PMC2713404

[3] Schindell, B. G., Webb, A. L. & Kindrachuk, J. Persistence and sexual transmission of filoviruses. *Viruses* **10**, 10.3390/v10120683 (2018). 10.3390/v10120683 PMC6316729 30513823

[4] Durant, O. & Marzi, A. Ebola virus disease sequelae and viral persistence in animal models: Implications for the future. *PLoS Pathog.* **20**, e1012065 (2024). 38512815 10.1371/journal.ppat.1012065 PMC10956775

[5] Anderson, E. M. & Coller, B. A. Translational success of fundamental virology: a VSV-vectored Ebola vaccine. *J. Virol.* e0162723. 10.1128/jvi.01627-23 (2024). 10.1128/jvi.01627-23 PMC10994820 38305150

[6] Tomori, O. & Kolawole, M. O. Ebola virus disease: current vaccine solutions. *Curr. Opin. Immunol.* **71**, 27–33 (2021). 33873076 10.1016/j.coi.2021.03.008

[7] Lee, A. Ansuvimab: first approval. *Drugs* **81**, 595–598 (2021). 33751449 10.1007/s40265-021-01483-4 PMC7983082

[8] Rayaprolu, V. et al. Structure of the Inmazeb cocktail and resistance to Ebola virus escape. *Cell Host Microbe* **31**, 260–272.e267 (2023). 36708708 10.1016/j.chom.2023.01.002 PMC10375381

[9] Mulangu, S., Mbala-Kingebeni, P. & Mbaya, O. T. Antibody use during an outbreak of Ebola virus disease in the Democratic Republic of Congo, 2020. *N. Engl. J. Med.* **386**, 1188–1191 (2022). 35320651 10.1056/NEJMc2113505

[10] Jones, S. M. et al. Live attenuated recombinant vaccine protects nonhuman primates against Ebola and Marburg viruses. *Nat. Med.* **11**, 786–790 (2005). 15937495 10.1038/nm1258

[11] Izudi, J. & Bajunirwe, F. Case fatality rate for Ebola disease, 1976–2022: a meta-analysis of global data. *J. Infect. Public Health* **17**, 25–34 (2024). 37992431 10.1016/j.jiph.2023.10.020

[12] Parish, L. A., Stavale, E. J., Houchens, C. R. & Wolfe, D. N. Developing vaccines to improve preparedness for filovirus outbreaks: the perspective of the USA biomedical advanced research and development authority (BARDA). *Vaccines* **11**, 10.3390/vaccines11061120 (2023). 10.3390/vaccines11061120 PMC10301178 37376509

[13] Marzi, A. et al. EBOLA VACCINE. VSV-EBOV rapidly protects macaques against infection with the 2014/15 Ebola virus outbreak strain. *Science* **349**, 739–742 (2015). 26249231 10.1126/science.aab3920 PMC11040598

[14] Henao-Restrepo, A. M. et al. Efficacy and effectiveness of an rVSV-vectored vaccine in preventing Ebola virus disease: final results from the Guinea ring vaccination, open-label, cluster-randomised trial (Ebola Ca Suffit!). *Lancet* **389**, 505–518 (2017). 28017403 10.1016/S0140-6736(16)32621-6 PMC5364328

[15] Marzi, A. et al. Single dose of a VSV-based vaccine rapidly protects macaques from marburg virus disease. *Front. Immunol.* **12**, 774026 (2021). 34777392 10.3389/fimmu.2021.774026 PMC8578864

[16] Marzi, A. & Feldmann, H. Ebola virus vaccines: an overview of current approaches. *Expert Rev. Vaccines* **13**, 521–531 (2014). 24575870 10.1586/14760584.2014.885841 PMC4785864

[17] Woolsey, C. et al. A recombinant vesicular stomatitis virus-based vaccine provides postexposure protection against bundibugyo ebolavirus infection. *J. Infect. Dis.* **228**, S712–S720 (2023). 37290053 10.1093/infdis/jiad207 PMC10651203

[18] Hunegnaw, R. et al. A single-shot ChAd3-MARV vaccine confers rapid and durable protection against Marburg virus in nonhuman primates. *Sci. Transl. Med.* **14**, eabq6364 (2022). 36516269 10.1126/scitranslmed.abq6364

[19] Lehrer, A. T. et al. Recombinant protein filovirus vaccines protect cynomolgus macaques from Ebola, Sudan, and Marburg Viruses. *Front. Immunol.* **12**, 703986 (2021). 34484200 10.3389/fimmu.2021.703986 PMC8416446

[20] Bockstal, V. et al. First-in-human study to evaluate safety, tolerability, and immunogenicity of heterologous regimens using the multivalent filovirus vaccines Ad26.Filo and MVA-BN-Filo administered in different sequences and schedules: a randomized, controlled study. *PloS ONE* **17**, e0274906 (2022). 36197845 10.1371/journal.pone.0274906 PMC9534391

[21] de La Vega, M. A. et al. An update on nonhuman primate usage for drug and vaccine evaluation against filoviruses. *Expert Opin. Drug Discov*. 10.1080/17460441.2024.2386100 (2024). 10.1080/17460441.2024.2386100 PMC11466704 39090822

[22] van Tol, S. et al. A bivalent Adenovirus-Vectored Vaccine induces a robust humoral response, but does not protect cynomolgus macaques against a lethal challenge with Sudan virus. *J. Infect. Dis.*10.1093/infdis/jiae056 (2024). 10.1093/infdis/jiae056 PMC11566226 38487996

[23] Kemenesi, G. et al. Isolation of infectious Lloviu virus from Schreiber’s bats in Hungary. *Nat. Commun.* **13**, 1706 (2022). 35361761 10.1038/s41467-022-29298-1 PMC8971391

[24] Bodmer, B. S. et al. In vivo characterization of the novel ebolavirus Bombali virus suggests a low pathogenic potential for humans. *Emerg. Microbes Infect.* **12**, 2164216 (2023). 36580440 10.1080/22221751.2022.2164216 PMC9858441

[25] Fletcher, P. et al. Pathogenicity of Lloviu and Bombali Viruses in Type I interferon receptor knockout mice. *J. Infect. Dis.* **228**, S548–S553 (2023). 37352146 10.1093/infdis/jiad226 PMC10651197

[26] Cross, R. W. et al. An introduction to the Marburg virus vaccine consortium, MARVAC. *PLoS Pathog.* **18**, e1010805 (2022). 36227853 10.1371/journal.ppat.1010805 PMC9560149

[27] Branswell, H. As foreign powers approve Ebola vaccines, U.S. drug makers lag in development pipeline. https://www.statnews.com/2017/12/08/ebola-vaccine-development/ (2017).

[28] WHO. *Ebola virus disease: Vaccines*, https://www.who.int/news-room/questions-and-answers/item/ebola-vaccines (2020).

[29] Merck. https://www.merck.com/news/u-s-fda-approves-mercks-ervebo-ebola-zaire-vaccine-live-for-use-in-children-12-months-of-age-and-older/, https://www.merck.com/news/european-commission-expands-mercks-ervebo-ebola-zaire-vaccine-rvsv%CE%B4gzebov-gp-live-indication-to-include-children-1-year-of-age-and-older/ (2023).

[30] WHO. *Four**countries in the African region license vaccine in milestone for Ebola prevention*, https://www.who.int/news/item/14-02-2020-four-countries-in-the-african-region-license-vaccine-in-milestone-for-ebola-prevention (2020).

[31] Kallay, R. et al. Use of Ebola vaccines - worldwide, 2021–2023. *MMWR Morb. Mortal. Wkly Rep.* **73**, 360–364 (2024). 38662631 10.15585/mmwr.mm7316a1 PMC11065462

[32] Coulborn, R. M. et al. Case fatality risk among individuals vaccinated with rVSVDeltaG-ZEBOV-GP: a retrospective cohort analysis of patients with confirmed Ebola virus disease in the Democratic Republic of the Congo. *Lancet Infect. Dis.*10.1016/S1473-3099(23)00819-8 (2024). 10.1016/S1473-3099(23)00819-8 38340736

[33] CDC. *Ebola (Ebola virus disease) - Outbreaks*, https://www.cdc.gov/vhf/ebola/history/chronology.html (2023).

[34] Nakkazi, E. A trial for Ebola Sudan virus in Uganda. *Lancet Infect. Dis.* **23**, 158 (2023). 36708723 10.1016/S1473-3099(23)00014-2

[35] Bwire, G. et al. Sudan Ebola virus (SUDV) outbreak in Uganda, 2022: lessons learnt and future priorities for sub-Saharan Africa. *BMC Med.* **21**, 144 (2023). 37055861 10.1186/s12916-023-02847-1 PMC10099013

[36] van Tol, S. et al. A bivalent Adenovirus-Vectored Vaccine induces a measurable humoral response, but does not protect cynomolgus macaques against a lethal challenge with Sudan virus. *bioRxiv.org*10.1101/2023.10.20.563337 (2023). 10.1093/infdis/jiae056 PMC11566226 38487996

[37] Honko, A. N. et al. A single-shot ChAd3 vaccine provides protection from intramuscular and aerosol sudan virus exposure. *bioRxiv*10.1101/2024.02.07.579118 (2024).

[38] Mwesigwa, B. et al. Safety, tolerability, and immunogenicity of the Ebola Sudan chimpanzee adenovirus vector vaccine (cAd3-EBO S) in healthy Ugandan adults: a phase 1, open-label, dose-escalation clinical trial. *Lancet Infect. Dis.* **23**, 1408–1417 (2023). 37544326 10.1016/S1473-3099(23)00344-4 PMC10837320

[39] Marzi, A. et al. Species-specific immunogenicity and protective efficacy of a vesicular stomatitis virus-based Sudan virus vaccine: a challenge study in macaques. *Lancet Microbe*10.1016/S2666-5247(23)00001-0 (2023). 10.1016/S2666-5247(23)00001-0 PMC10010116 36739878

[40] Geisbert, T. W. et al. Single-injection vaccine protects nonhuman primates against infection with marburg virus and three species of ebola virus. *J. Virol.* **83**, 7296–7304 (2009). 19386702 10.1128/JVI.00561-09 PMC2704787

[41] To, A. et al. Thermostable bivalent filovirus vaccine protects against severe and lethal Sudan ebolavirus and marburgvirus infection. *Vaccine* **42**, 598–607 (2024). 38158300 10.1016/j.vaccine.2023.12.053 PMC10872277

[42] Towner, J. S. et al. Newly discovered ebola virus associated with hemorrhagic fever outbreak in Uganda. *PLoS Pathog.* **4**, e1000212 (2008). 19023410 10.1371/journal.ppat.1000212 PMC2581435

[43] MacNeil, A. et al. Proportion of deaths and clinical features in Bundibugyo Ebola virus infection, Uganda. *Emerg. Infect. Dis.* **16**, 1969–1972 (2010). 21122234 10.3201/eid1612.100627 PMC3294552

[44] Kratz, T. et al. Ebola virus disease outbreak in Isiro, Democratic Republic of the Congo, 2012: signs and symptoms, management and outcomes. *PloS ONE* **10**, e0129333 (2015). 26107529 10.1371/journal.pone.0129333 PMC4479598

[45] Falzarano, D. et al. Single immunization with a monovalent vesicular stomatitis virus-based vaccine protects nonhuman primates against heterologous challenge with Bundibugyo ebolavirus. *J. Infect. Dis.* **204**, S1082–S1089 (2011). 21987745 10.1093/infdis/jir350 PMC3189995

[46] Mire, C. E. et al. Vesicular stomatitis virus-based vaccines protect nonhuman primates against Bundibugyo ebolavirus. *PLoS Neglect. Trop. Dis.* **7**, e2600 (2013). 10.1371/journal.pntd.0002600 PMC3868506 24367715

[47] Formenty, P. et al. Human infection due to Ebola virus, subtype Cote d’Ivoire: clinical and biologic presentation. *J. Infect. Dis.* **179**, S48–S53 (1999). 9988164 10.1086/514285

[48] CDC. *Marburg Disease Outbreaks*, https://www.cdc.gov/vhf/marburg/outbreaks/chronology.html (2023).

[49] Marzi, A. et al. Vesicular stomatitis virus-based Ebola vaccines with improved cross-protective efficacy. *J. Infect. Dis.* **204**, S1066–S1074 (2011). 21987743 10.1093/infdis/jir348 PMC3203393

[50] Fletcher, P. et al. Single-dose VSV-based vaccine protects cynomolgus macaques from disease after Tai Forest virus infection. *Emerg. Microbes Infect.* **12**, 2239950 (2023). 37470396 10.1080/22221751.2023.2239950 PMC10392270

[51] Callendret, B. et al. A prophylactic multivalent vaccine against different filovirus species is immunogenic and provides protection from lethal infections with Ebolavirus and Marburgvirus species in non-human primates. *PloS ONE* **13**, e0192312 (2018). 29462200 10.1371/journal.pone.0192312 PMC5819775

[52] Tiemessen, M. M. et al. Protection against Marburg virus and Sudan virus in NHP by an adenovector-based trivalent vaccine regimen is correlated to humoral immune response levels. *Vaccines* **10**, 10.3390/vaccines10081263 (2022). 10.3390/vaccines10081263 PMC9412258 36016151

[53] Slenczka, W. & Klenk, H. D. Forty years of Marburg virus. *J. Infect. Dis.* **196**, S131–S135 (2007). 17940940 10.1086/520551

[54] Woolsey, C. et al. A highly attenuated Vesiculovax vaccine rapidly protects nonhuman primates against lethal Marburg virus challenge. *PLoS Neglect. Trop. Dis.* **16**, e0010433 (2022). 10.1371/journal.pntd.0010433 PMC9182267 35622847

[55] Marzi, A. et al. Protection against Marburg virus using a recombinant VSV-vaccine depends on T and B cell activation. *Front. Immunol.* **9**, 3071 (2018). 30723475 10.3389/fimmu.2018.03071 PMC6350103

[56] O’Donnell, K. L. et al. Rapid protection of nonhuman primates against Marburg virus disease using a single low-dose VSV-based vaccine. *EBioMedicine* **89**, 104463 (2023). 36774693 10.1016/j.ebiom.2023.104463 PMC9947254

[57] Mire, C. E. et al. Durability of a vesicular stomatitis virus-based Marburg virus vaccine in nonhuman primates. *PloS ONE* **9**, e94355 (2014). 24759889 10.1371/journal.pone.0094355 PMC3997383

[58] Hamer, M. J. et al. Safety, tolerability, and immunogenicity of the chimpanzee adenovirus type 3-vectored Marburg virus (cAd3-Marburg) vaccine in healthy adults in the USA: a first-in-human, phase 1, open-label, dose-escalation trial. *Lancet* **401**, 294–302 (2023). 36709074 10.1016/S0140-6736(22)02400-X PMC10127441

[59] Matassov, D. et al. Single-dose trivalent VesiculoVax vaccine protects macaques from lethal ebolavirus and marburgvirus challenge. *J. Virol.* **92**, 10.1128/JVI.01190-17 (2018). 10.1128/JVI.01190-17 PMC5774882 29142131

[60] Cross, R. W. et al. Quadrivalent VesiculoVax vaccine protects nonhuman primates from viral-induced hemorrhagic fever and death. *J. Clin. Invest.* **130**, 539–551 (2020). 31820871 10.1172/JCI131958 PMC6934204

[61] Woolsey, C. et al. A highly attenuated panfilovirus VesiculoVax vaccine rapidly protects nonhuman primates against marburg virus and 3 species of Ebola virus. *J. Infect. Dis.* **228**, S660–S670 (2023). 37171813 10.1093/infdis/jiad157 PMC11009496

[62] Marzi, A., Feldmann, H., Geisbert, T. W. & Falzarano, D. Vesicular stomatitis virus-based vaccines for prophylaxis and treatment of filovirus infections. *J. Bioterrorism Biodefense* **S1**, 10.4172/2157-2526.S1-004 (2011). 10.4172/2157-2526.S1-004 PMC3265573 22288023

[63] Mire, C. E. et al. Oral and conjunctival exposure of nonhuman primates to low doses of Ebola Makona Virus. *J. Infect. Dis.* **214**, S263–S267 (2016). 27284090 10.1093/infdis/jiw149 PMC5050459

[64] Prasad, A. N. et al. Natural history of nonhuman primates after oral exposure to Ebola Virus Variant Makona. *J. Infect. Dis.* **228**, S571–S581 (2023). 37348509 10.1093/infdis/jiad225 PMC10651204

[65] Reynolds, P. & Marzi, A. Ebola and Marburg virus vaccines. *Virus Genes* **53**, 501–515 (2017). 28447193 10.1007/s11262-017-1455-x PMC7089128

